# Development of a Neural Network to Predict Optimal IOP Reduction in Glaucoma Management

**DOI:** 10.3390/vision9040087

**Published:** 2025-10-15

**Authors:** Raheem Remtulla, Sidrat Rahman, Hady Saheb

**Affiliations:** 1 McGill Academic Eye Centre, McGill University, Montreal, QC H4A 3J1, Canada; raheem.remtulla@mail.mcgill.ca; 2 Faculty of Medicine, McGill University, Montreal, QC H4A 3J1, Canada; 3 McGill University Health Centre—Research Institute, McGill University, Montreal, QC H4A 3J1, Canada

**Keywords:** neural network, glaucoma management, feature importance, precision ophthalmology

## Abstract

Glaucoma management relies on lowering intraocular pressure (IOP), but determining the target reduction at presentation is challenging, particularly in normal-tension glaucoma (NTG). We developed and internally validated a neural network regression model using retrospective clinical data from Qiu et al. (2015), including 270 patients (118 with NTG). A single-layer artificial neural network with five nodes was trained in MATLAB R2024b using the Levenberg–Marquardt algorithm. Inputs included demographic, refractive, structural, and functional parameters, with IOP reduction as the output. Data were split into 65% training, 15% validation, and 20% testing, with training repeated 10 times. Model performance was strong and consistent (average RMSE: 1.90 ± 0.29 training, 2.18 ± 0.34 validation, 2.11 ± 0.30 testing; Pearson’s r: 0.92 ± 0.02, 0.88 ± 0.02, 0.88 ± 0.04). The best-performing model achieved RMSEs of 1.57, 2.90, and 1.77 with r values of 0.93, 0.91, and 0.93, respectively. Feature ablation revealed significant contributions from IOP, axial length, CCT, diagnosis, VCDR, spherical equivalent, mean deviation, and laterality. This study demonstrates that a simple neural network can reliably predict individualized IOP reduction targets, supporting personalized glaucoma management and improved outcomes.

## 1. Introduction

Glaucoma is a chronic eye disease and a leading cause of irreversible blindness worldwide, particularly in individuals over the age of 60 [1]. It is a progressive condition caused by damage to the optic nerve, ultimately resulting in visual field loss [2]. Primary open-angle glaucoma (POAG) and normal-tension glaucoma (NTG) are among the most common subtypes, both of which typically respond to intraocular pressure (IOP)-lowering therapies [3].

Landmark glaucoma trials have highlighted the importance of IOP reduction in slowing disease progression. For example, the EMGT demonstrated that a 25% reduction in IOP significantly delays POAG progression, while the CNTGS showed that a 30% reduction similarly slows progression in NTG [4,5,6]. However, determining the optimal IOP reduction for individual patients remains challenging and is often ophthalmologist-dependent. Current clinical guidelines recommend an initial IOP reduction of 20–30%, followed by ongoing monitoring of the optic nerve and visual field function to guide adjustments [7,8].

Despite these strategies, some patients continue to progress even after achieving target IOP levels [5,9,10]. This can be particularly problematic in NTG, where baseline IOP levels fall within normal population ranges, making it difficult to determine and achieve the extent of additional reduction required [3,11]. Consequently, there is a critical need for predictive tools to individualize treatment and improve long-term outcomes.

The application of artificial intelligence (AI) through neural networks demonstrates potential to enhance clinical decision-making in ophthalmic care, especially when treating glaucoma patients [12,13,14]. Neural networks are well suited for analyzing complex diseases like glaucoma because they can identify hidden patterns across multiple clinical variables. Recent research has highlighted the value of AI in improving diagnosis and expanding access to care in ophthalmology, particularly for conditions such as glaucoma and diabetic retinopathy. Building on these findings, AI-based models that integrate ocular biometry, visual field data, refractive error, and optic nerve features may offer a more accurate and personalized approach to determining intraocular pressure targets than current empirical methods [15]. This method could streamline decision-making, decrease the need for multiple therapeutic adjustments, and ultimately improve patient outcomes. By estimating the optimal IOP reduction at presentation, a neural network could enable earlier, individualized treatment strategies and reduce reliance on trial-and-error therapy adjustments.

In this study, we aimed to develop and validate a single-layer neural network capable of predicting the required IOP reduction at initial presentation for both POAG and NTG patients. These factors, including both ocular and systemic variables, were analyzed, and the model’s predictive performance carefully evaluated. Additionally, we conducted a broad feature importance analysis by systematically removing each input parameter and assessing the resulting impact on model performance. The goal was to provide clinicians with a safe and reliable tool to guide early therapeutic decisions and to better understand the relative importance of specific risk factors that influence IOP management strategies.

## 2. Materials and Methods

Data were obtained from the article by Qiu et al. (2015) titled “Axial Myopia Is Associated with Visual Field Prognosis of Primary Open-Angle Glaucoma,” involving 270 patients, of whom 118 had NTG [16]. Patients with POAG were included if they had over three years of follow-up, at least nine reliable VF tests, and received medical treatment. Patients were excluded if they had refractive errors with a SE less than −9.0 D or greater than +6.0 D, a history of intraocular, refractive, or laser surgery, BCVA less than 20/40, VF mean defect worse than −20 dB, or any ocular or neurological conditions that could interfere with accurate assessment of the optic nerve or visual field. The dataset from Qiu et al. (2015) [16], while robust in its clinical follow-up and visual field data, was not originally designed for predictive modeling of therapeutic outcomes. Its retrospective nature and single-center origin may limit generalizability and introduce bias, particularly given potential confounders not captured in the dataset. Future work should incorporate prospectively collected, multi-center data with broader clinical and demographic features to validate the model.

**Figure 1:**
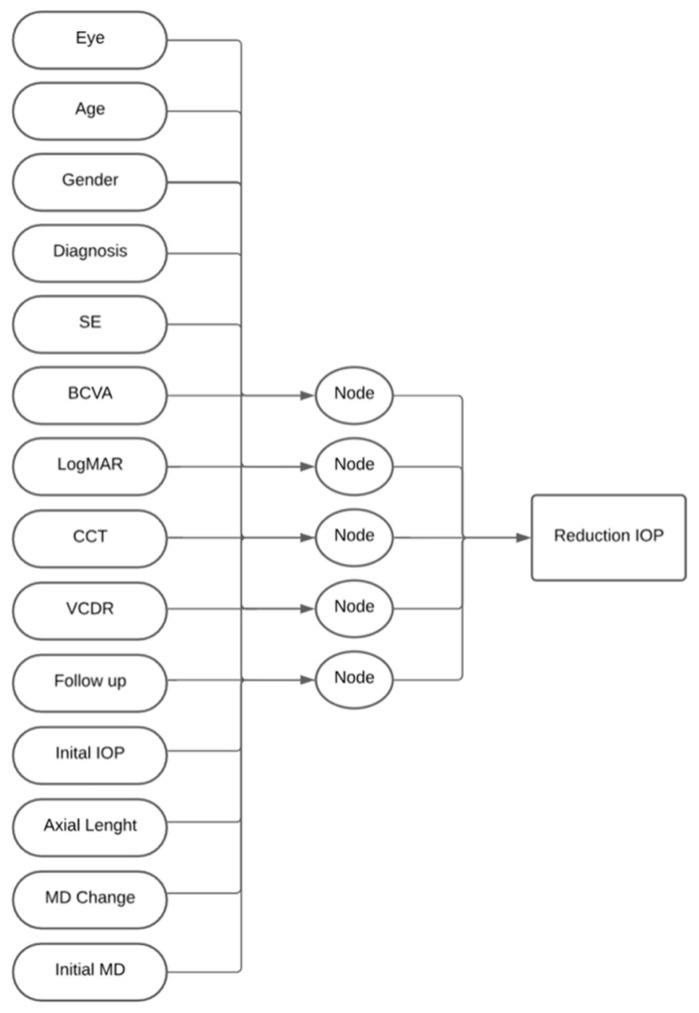
Network architecture demonstrating single-layer artificial neural network with five nodes. Inputs included eye laterality, age, gender, glaucoma subtype, spherical equivalence, best-corrected and LogMAR visual acuity, central corneal thickness, vertical cup-to-disk ratio, follow-up duration, baseline IOP, axial length, and visual field metrics.

A standard artificial neural network was constructed using the Levenberg–Marquardt methodology in MATLAB R2024b’s Neural Network Start tool (MathWorks, Inc., Santa Clara, CA, USA) [17]. The network consisted of a single layer with five nodes, as illustrated in Figure 1. The dataset was split into 65% for training, 15% for validation, and 20% for testing. Input factors included eye laterality, age, gender, diagnosis (POAG vs. NTG), spherical equivalence (SE), best-corrected visual acuity (BCVA), LogMAR BCVA, central corneal thickness (CCT), vertical cup-to-disk ratio (VCDR), follow-up timeline, axial length, initial mean deviation (MD) on visual field, and total change in MD during follow-up period. The target output was the IOP reduction over the follow-up timeframe. To evaluate model robustness and potential data fragility, the complete training process was repeated ten times using different random seeds. Each iteration preserved the same 65/15/20% data split. Average and range values for RMSE and Pearson’s r were computed across all runs to assess performance consistency and resistance to random partition effects. Network performance was measured by root mean squared error (RMSE) and Pearson’s r. The network was implemented in MATLAB R2024b (feedforwardnet, 5 units; trainlm; MSE objective), with inputs z-score normalized (mapstd), data divided by dividerand [0.65, 0.15, 0.20], and early stopping on the validation set (max_fail = 6). Fixed random seeds (rng 42–51) were used for the 10 repeats, and the same pipeline was applied for baseline and ablation runs to ensure reproducibility.

To evaluate the contribution of each input variable to model performance, we conducted a leave-one-feature-out (LOFO) ablation analysis [18,19]. A baseline neural network was trained using the 12 clinical input features available at first presentation, and its performance was assessed using the RMSE and Pearson’s r [20,21]. To ensure stability and account for variability in training, each model configuration was trained 10 times, and the performance metrics were averaged. The model was then retrained iteratively, each time excluding one feature, with the same 10-run averaging approach. The resulting changes in average RMSE, relative to the baseline, were used to quantify the importance of each feature. This method provides a robust, model-specific estimate of feature relevance by directly measuring the impact of individual variables on predictive accuracy. It also aligns with established practices in feature attribution and model interpretation, offering a transparent approach to understanding model behavior [22,23,24,25]. Statistical comparisons between baseline and ablation models were conducted using a one-tailed paired Student’s *t*-test to determine whether feature removal resulted in a significant increase in RMSE.

## 3. Results

The average performance across all 10 networks demonstrated that the model could effectively predict the patient IOP reduction from presentation. The average RMSE values indicated appropriate predictive ability, with averages of 1.90 ± 0.29 for the training set, 2.18 ± 0.34 for the validation set, and 2.11 ± 0.30 for the testing set. These results suggest that the model maintained a consistent and reliable performance across all datasets with minimal overfitting. The Pearson’s r correlation coefficient values further supported the model’s effectiveness, with averages of 0.92 ± 0.02 for training, 0.88 ± 0.02 for validation, and 0.88 ± 0.04 for testing. Performance remained stable across all random seeds, indicating low data fragility and confirming that the model’s predictive behavior was not dependent on a single initialization or data split. A detailed summary of these performance metrics is presented in Table 1.

**Figure 2:**
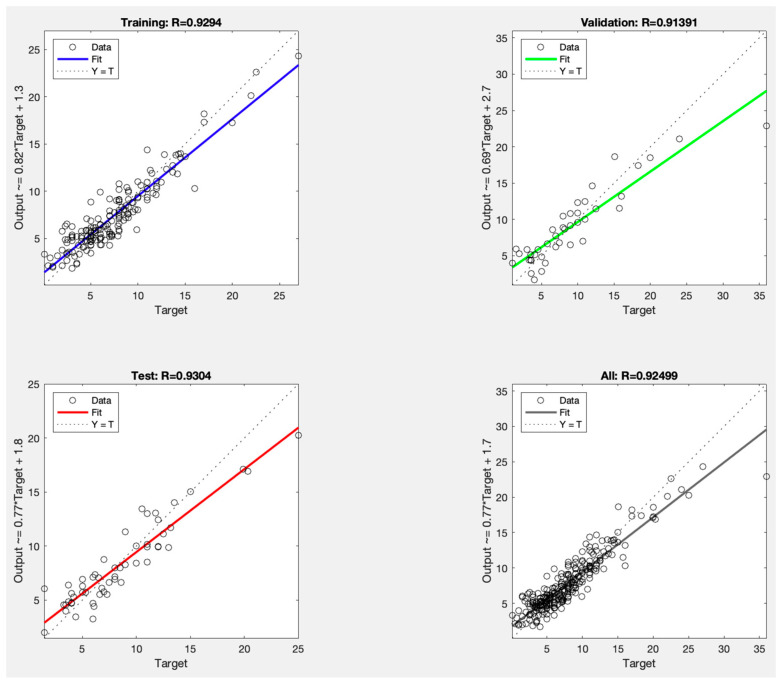
Calibration plot for model performance across data subsets of best-performing neural network.

**Figure 3:**
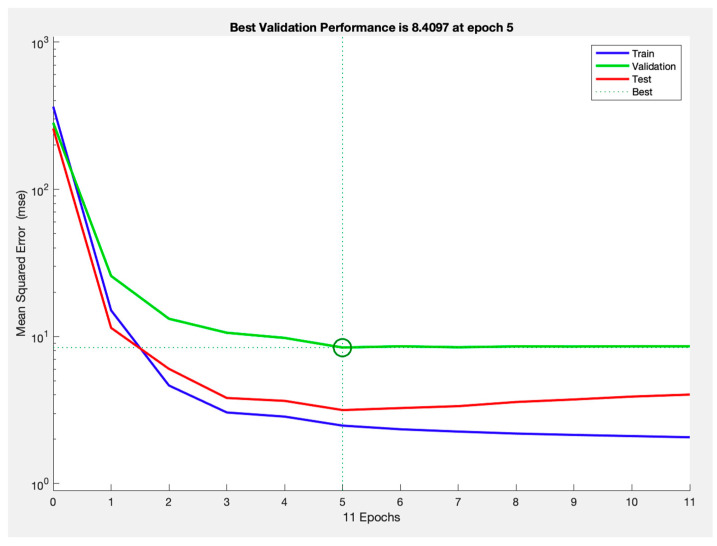
Performance of best-performing neural network through epochs of training.

The best-performing network had an RMSE of 1.57 for training, 2.90 for validation, and 1.77 for testing. The corresponding r were 0.93 for training, 0.91 for validation, and 0.93 for testing, indicating accuracy in predicting IOP reduction. A detailed summary of these performance metrics is presented in Table 2 and Figure 2. Notably, optimal performance was achieved after 11 epochs, as illustrated in Figure 3.

LOFO analysis was performed to assess the relative importance of individual input variables. This approach involved systematically retraining the model while excluding one feature at a time and evaluating the impact on predictive performance using RMSE. Statistically significant increases in RMSE were observed upon removal of initial intraocular pressure (IOP), axial length, central corneal thickness (CCT), glaucoma diagnosis, vertical cup-to-disk ratio (VCDR), spherical equivalence, mean deviation, and eye laterality (see Table 3). These findings suggest that these features play a critical role in model accuracy, while the remaining features did not contribute significantly to performance degradation when excluded.

## 4. Discussion

The neural network developed in this study was trained to predict the expected reduction in IOP using 13 clinical and ocular features, including eye laterality, age, gender, glaucoma subtype, spherical equivalence, best-corrected and LogMAR visual acuity, central corneal thickness, vertical cup-to-disk ratio, follow-up duration, baseline IOP, axial length, and visual field metrics (initial mean deviation and its change over time). A key potential application of this model lies in the advancement of personalized medicine. By allowing clinicians to set a patient-specific, tolerable threshold for visual field progression, reflected as a target change in mean deviation, the model can be used in reverse to estimate the IOP reduction necessary to achieve that outcome. This approach facilitates individualized IOP target setting aligned with each patient’s disease characteristics, risk tolerance, and visual goals, ultimately supporting more patient-centered glaucoma care.

The best-performing network achieved a testing RMSE of 1.77 and a Pearson’s r correlation coefficient of 0.93, confirming that a single-layer neural network with limited complexity can effectively capture meaningful patterns within the dataset. The low RMSE and high Pearson’s r correlation coefficients observed across the training, validation, and testing sets demonstrate strong predictive accuracy and minimal overfitting. By providing an immediate, patient-specific IOP goal, clinicians could potentially move away from a purely reactive approach and achieve a more proactive, data-driven management strategy.

Current practice often involves initiating IOP-lowering therapy and adjusting targets in response to observed progression, a process which can result in both over- and under-treatment. This over- or under-treatment risk is related to the difference between the clinician’s expectation of the patient’s glaucoma journey and the patient’s actual journey, which are not always aligned. Overly aggressive IOP-lowering may increase the burden of therapy, raise the risk of adverse effects, impose the risks of unnecessary surgery, and reduce patient quality of life [23]. Conversely, insufficient IOP reduction may fail to halt disease progression in a timely manner. A predictive model that accounts for individualized thresholds can help refine initial treatment targets. This approach is especially valuable in cases of NTG, where determining an adequate pressure goal remains particularly challenging [3,6].

The LOFO analysis revealed that several input features traditionally associated with glaucoma progression, such as spherical equivalence and axial length (markers of myopia), baseline IOP, VCDR, glaucoma subtype, initial mean deviation MD, and central corneal thickness CCT, were identified as important contributors to model performance [26,27,28]. For example, thinner CCT and larger VCDR have been associated with greater risk of progression [22,29]. Similarly, the role of axial length and refractive error in glaucoma susceptibility has been increasingly recognized, as longer axial lengths can be linked to changes in the biomechanical environment of the optic nerve head [30]. This alignment with known clinical predictors provides strong construct validity for the neural network and offers reassurance that the model is not functioning as a purely opaque “black box,” but rather is learning physiologically and clinically meaningful relationships [22,24,25]. Conversely, features not classically associated with glaucoma progression, including gender and visual acuity, were not found to significantly impact model accuracy in the LOFO analysis. Their limited influence on network performance further supports the model’s alignment with established clinical knowledge and adds to its construct validity.

Interestingly, age, despite being a recognized risk factor for glaucoma progression, did not emerge as an important feature in the LOFO evaluation. One possible explanation is that clinicians may tolerate higher IOP levels in older patients based on the understanding that glaucomatous damage may not cause meaningful visual impairment within the patient’s remaining lifespan. This clinical judgment likely introduced confounding into the training data, particularly among patients with more advanced disease who were assigned higher IOP targets. Another unexpected finding was that eye laterality had a statistically significant effect on RMSE when excluded from the model, although it had the smallest RMSE increase among the significant features. There is no established literature supporting a role for eye laterality in glaucoma progression. One speculative explanation is that subtle and multifactorial processes may contribute to asymmetric progression in an otherwise bilateral disease. However, this remains unlikely and is not currently supported by evidence. More plausibly, the importance of eye laterality in this analysis reflects limitations in dataset diversity and sample size. A fully functional and clinically applicable network will likely require a larger, more heterogeneous patient cohort to improve generalizability.

Of the 12 features included in the LOFO analysis, 10 showed results that were consistent with current clinical understanding. This suggests that the neural network was generally able to identify key features associated with glaucomatous progression, providing further construct validity and confidence in the model’s potential for future clinical use.

Taken together, our model demonstrates both the promise and limitations of our model. There are several factors in our study that warrant consideration. While the sample size is reasonable, expanding it to include more diverse populations and various subtypes of glaucoma could strengthen the findings. A larger dataset would not only enable more robust validation but also enhance the model’s generalizability. Moreover, prior research has consistently highlighted the limited reproducibility and poor interrater reliability of human gradings, further underscoring the need for improved methodologies [26,27,28,31].

Incorporating genetic information could significantly enhance the predictive power of neural networks modeling IOP reduction in glaucoma patients. Genetic variants such as those in MYOC, OPTN, and CAV1/CAV2 have been associated with glaucoma risk, treatment response, and baseline IOP levels [32,33]. For instance, polymorphisms in CYP1B1 and LTBP2 have shown associations with differential responses to prostaglandin analogs and beta-blockers [34,35]. By integrating such genetic markers into the model, the network could account for inter-individual variability in pharmacodynamics and disease progression, potentially enabling more personalized and effective treatment strategies. Future development of these neural networks should also aim to incorporate longitudinal imaging, advanced ocular biometric data, and relevant systemic factors that influence glaucoma progression.

One limitation of the current model is its tendency to overestimate the IOP reduction necessary to prevent glaucomatous progression. Therefore, the authors introduce the speculative concept of a Zero IOP-induced Nerve Ganglionopathy (ZING) level, the threshold beyond which further IOP reduction yields no additional therapeutic benefit. In any given patient, once IOP is reduced below their personal ZING threshold, additional reduction may not confer further benefit in halting disease progression. However, because the model was trained on observed clinical data, cases in which patients achieved IOP reductions greater than their ZING level but showed no further visual field decline were interpreted by the model as requiring that same degree of reduction. As a result, the network may generalize these larger-than-necessary IOP reductions as the required target, potentially overestimating the ideal therapeutic goal. This limitation underscores the value of incorporating individualized thresholds such as ZING and applying clinical reasoning to improve the precision of predictive modeling in glaucoma care. ZING represents a hypothesis-generating concept that has not yet been empirically validated. The concept is presented to stimulate further experimental investigation. No prior studies have explicitly defined a “no-benefit IOP threshold” in glaucoma progression. However, the idea aligns with the broader recognition that glaucoma is a multifactorial neurodegenerative disease and that additional lowering of IOP does not always prevent progression; some patients continue to lose ganglion cells despite apparently adequate pressure control, a phenomenon consistent with residual risk from non-pressure mechanisms [36]. Future research should prospectively examine patient cohorts across wide IOP ranges to estimate potential ZING levels using longitudinal structural and functional endpoints to evaluate its validity.

A key limitation of this study is the absence of external validation. The model was trained and tested using a single-center retrospective dataset, which may restrict its generalizability to other populations, refractive profiles, and clinical environments. In addition, the number of NTG cases was relatively limited, which may reduce the model’s ability to fully capture the variability within this subgroup. Nevertheless, performance consistency across multiple training iterations suggests that the available NTG data contributed stably to model learning. Future research will focus on validating the neural network using multi-center, multi-ethnic datasets and independent cohorts to confirm its robustness and transportability across different clinical settings.

Implementing this type of predictive model in routine clinical practice requires careful integration. Clinicians must learn to interpret model outputs alongside traditional risk assessments, aided by clear guidelines and training resources that translate predicted IOP targets into informed therapeutic decisions. Through external validation across diverse populations and practice settings will help confirm reproducibility, reduce biases, and further enhance its utility [30]. Ensuring high-quality data, including glaucoma subtypes and relevant patient characteristics, and strictly upholding ethical standards such as patient privacy will be essential. These efforts will support the meaningful integration of AI models as medicine continues to move toward data-driven and personalized care [37].

Clinical implementation of the model would ideally occur within a decision-support framework, where patient-specific demographic, structural, and functional parameters are entered into an interface integrated with the electronic medical record. The model’s output could provide a data-informed range for target intraocular pressure (IOP) reduction, accompanied by performance metrics or uncertainty indicators to support physician interpretation. Such an approach would enable stratification of patients according to the predicted degree of IOP-lowering required, guiding the intensity of treatment while preserving clinical oversight. Specification of strict upper and lower bounds for model-recommended IOP reduction is not currently feasible, as these limits are data-dependent and influenced by prediction accuracy across the observed range. Uncertainty increases at extreme values where data are sparse, and excessive IOP-lowering may introduce clinical risk. The model’s output should therefore be interpreted as a flexible, data-driven range rather than a fixed target. Future studies using larger, multi-center datasets may refine these limits and improve generalizability.

A pragmatic pathway to clinical implementation involves a prospective, random comparison of model-guided IOP targets versus standard care. The model-guided arm would deliver a data-informed target range with safety guardrails aligned to institutional standards and the observed data distribution; the control arm would use clinician-determined targets. Visual field progression would serve as the primary endpoint, with treatment burden, surgical intervention rates, structural progression, patient-reported outcomes, and safety as secondary endpoints. Integration into routine care would occur through EMR embedding with structured inputs and automated data pull; physician training would be delivered via brief competency modules and in-app examples; interpretability would be supported by display of key input contributions, sensitivity summaries, and uncertainty indicators; liability would be addressed by positioning the system as decision support with final decisions retained by the clinician, audit trails, and documentation when deviating from recommendations.

Practical challenges include heterogeneous data formats, missing or low-quality inputs, distribution shift, and performance variation across subgroups. Feasible solutions include standardized data schemas and quality checks at ingestion, default fallback rules for incomplete inputs, post-deployment monitoring with scheduled recalibration, site-level validation before broader rollout, and bias audits with predefined mitigation plans. A phased deployment with pilot clinics, user-centered interface testing, and change-management support would enable safe adoption without disrupting workflows.

By integrating comprehensive ophthalmic data, demographic informainstion, and established risk factors for glaucomatous progression, our neural network model provides a personalized and clinically relevant approach to glaucoma management. Its consistent predictive performance across training, validation, and testing phases underscores its reliability and potential utility in practice. When properly trained and validated, such AI-driven tools can offer precise predictions for target IOP reduction, enabling clinicians to tailor treatment strategies from the outset and enhance patient outcomes. Beyond guiding individualized care, feature importance analysis offers valuable insights into the factors influencing disease progression, highlighting the potential of these innovations to advance precision medicine and transform ophthalmic care.

## Figures and Tables

**Table 1:** Average network performance across all 10 networks trained. RMSE and r for the training, validation, and test sets are provided.

	RMSE Average	r
Training	1.90 ± 0.29	0.92 ± 0.02
Validation	2.18 ± 0.34	0.88 ± 0.02
Test	2.11 ± 0.30	0.88 ± 0.04

**Table 2:** Network performance for the best-trained neural network. RMSE and r for the training, validation, and test sets are provided.

	RMSE	r
Training	1.57	0.93
Validation	2.90	0.91
Test	1.77	0.93

**Table 3:** Statistically significant increases in test-set RMSE observed only for input factors that reached significance following removal from the base neural network.

	RMSE	RMSE Increase	*p*-Value
Initial IOP	4.56 ± 0.96	2.45	<0.001
Axial length	2.74 ± 0.52	0.63	<0.01
CCT	2.65 ± 0.34	0.54	<0.001
Glaucoma Diagnosis	2.63 ± 0.67	0.52	0.04
VCDR	2.59 ± 0.49	0.48	0.01
SE	2.58 ± 0.47	0.47	<0.01
MD Data	2.43 ± 0.38	0.32	0.04
Eye laterality	2.38 ± 0.41	0.27	0.02

## Data Availability

The original contributions presented in this study are included in the article. Further inquiries can be directed to the corresponding author.

## Abbreviations

The following abbreviations are used in this manuscript:

BCVA	Best-Corrected Visual Acuity
CAV1/CAV2	Caveolin 1/Caveolin 2
CCT	Central Corneal Thickness
CNTGS	Collaborative Normal-Tension Glaucoma Study
CYP1B1	Cytochrome P450 Family 1 Subfamily B Member 1
EMGT	Early Manifest Glaucoma Trial
IOP	Intraocular Pressure
LogMAR	Logarithm of the Minimum Angle of Resolution
LOFO	Leave-One-Feature-Out
LTBP2	Latent Transforming Growth Factor Beta Binding Protein 2
MD	Mean Deviation (on Visual Field)
MYOC	Myocilin
NTG	Normal-Tension Glaucoma
OPTN	Optineurin
POAG	Primary Open-Angle Glaucoma
RMSE	Root Mean Squared Error
SE	Spherical Equivalence
VF	Visual Field
VCDR	Vertical Cup-to-Disk Ratio
ZING	Zero IOP-Induced Nerve Ganglionopathy
